# A DNA Recombination “Hotspot” in Humans Is Missing in Chimps

**DOI:** 10.1371/journal.pbio.0020192

**Published:** 2004-06-15

When Francis Collins and Craig Venter reported the draft sequence of the human genome in 2001, Collins described the so-called book of life as more of a life sciences encyclopedia. In it, we can find our evolutionary history written in the fossil record of our DNA, a parts manual listing the genes and proteins needed to build and operate a human being, and a medical text, gleaned from the genetic variants linked to human disease. Unfortunately, he added, the texts are written in a language “we don't entirely know how to read yet.” Since then, biologists have made great progress in extracting meaning from the human genome. Humans are 99.9% alike genetically, and that 0.1% makes all the difference in terms of appearance, personality, and susceptibility to disease. That 0.1% promises to shed light on the evolutionary forces that control genetic variation as well as the genetic origins of human disease.[Fig pbio-0020192-g001]

**Figure pbio-0020192-g001:**
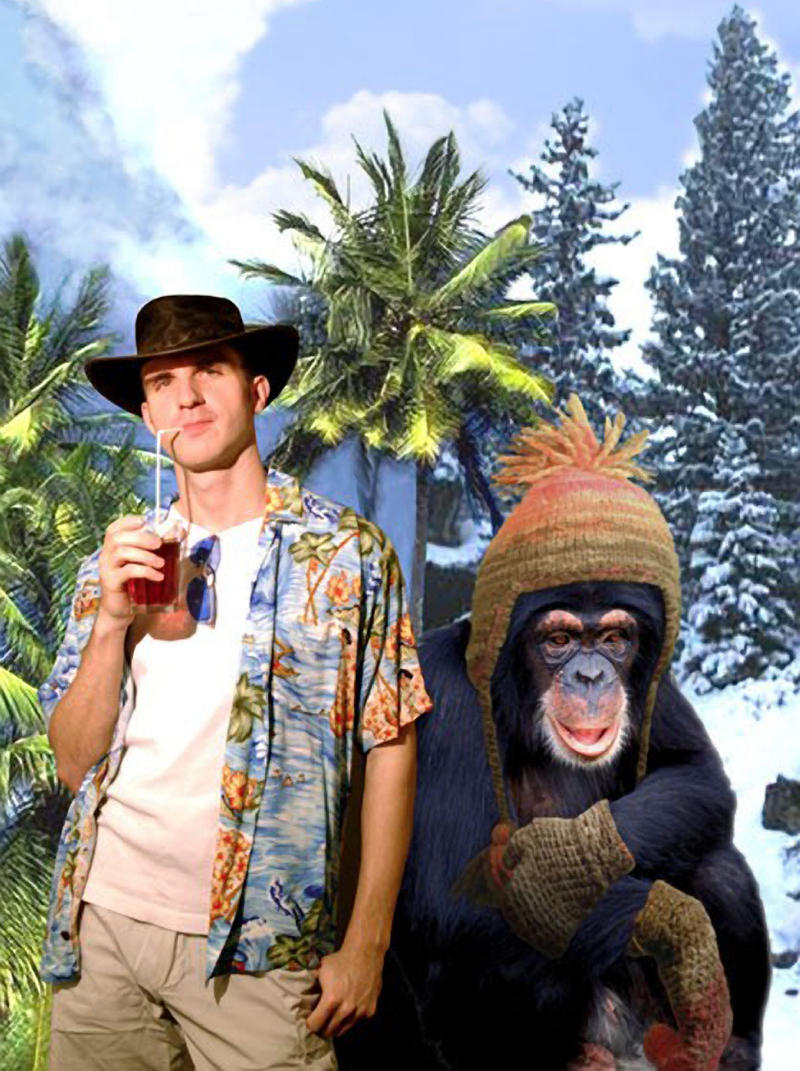
The TAP2 region harbors a recombination hotspot in humans. What about in chimpanzees: hot or cold?

Very small genetic variations—including differences of a single DNA base, called single nucleotide polymorphisms, or SNPs—occur through random mutations. Individuals have two of each chromosome (one from the mother and one from the father), and the combination of SNPs found together on one chromosome can change through the random shuffling of genetic material between the two chromosomes when sperm and egg cells are produced during meiosis. By studying the location and frequency of this reassortment in the genome, biologists hope to understand how recombination affects the overall pattern of SNP variation and how these patterns relate to human disease. An international collaborative effort called the HapMap Project aims to identify the most common SNP associations within chromosomes, known as haplotypes, and then determine which haplotypes are associated with disease. This approach relies on what's known as “linkage disequilibrium”—the nonrandom association of alleles (gene variants) at different locations on a chromosome—to facilitate their search for candidate disease genes. Adjacent SNPs show strong linkage disequilibrium, which means that researchers can select a limited number of SNPs as markers for a haplotype and test their association with disease rather than testing each SNP.

Patterns of linkage disequilibrium depend on the rate of recombination—higher recombination rates typically cause less linkage. Demographic factors and chance also affect levels of linkage disequilibrium; while both vary across populations, it has been thought that recombination rates do not. Recombination appears to favor specific genomic regions, termed hotspots, but the observation that the recombinant chromosomes are not passed down in equal proportions suggests that recombination hotspots may be short-lived, appearing as transient blips on the evolutionary radar. Exploring this possibility, Susan Ptak et al. compared a well-studied recombination hotspot in humans, called TAP2, with a similar region in our closest evolutionary cousins, chimpanzees, to see whether they are similarly endowed.

Since recombination occurs relatively rarely, researchers have relied largely on indirect methods to determine regional recombination rates. Though recent advances have made sperm analysis in humans more practical (though still technically challenging), such techniques are less feasible with chimps because collecting large amounts of sperm from individual males might compromise their success in mating competition or reduce the genetic diversity of endangered chimp populations. Here, the researchers used an indirect approach to estimate recombination rates from the patterns of linkage distribution, which “reflect the rate and distribution of recombination events in the ancestors of the sample.” They focused on chimps from a single subspecies because the reported high level of genetic differentiation between subspecies could skew estimates of recombination rate variation. Analysis of the TAP2 region revealed 47 SNPs in the human and 57 in the chimp, with an overall lower level of linkage disequilibrium in humans: strong linkage was seen only in adjacent pairs of SNPs in humans, but was found in both adjacent and more distant pairs in the chimps. Using a statistical approach to characterize recombination rate variation between the two species, Ptak et al. found “extremely strong support” for rate variation in humans but found strong evidence against such variation in chimps.

Humans and chimps diverged from a common ancestor five to six million years ago and differ at only 1.2% of base pairs on average. That the recombination hotspot does not exist in both species suggests that hotspots are not stable and can evolve fairly quickly. If recombination rates within a small genomic area—at the level of a few thousand bases—can change in such a short time frame between such closely related species, Ptak et al. reason, they may do so within species, too. Such a prospect has important implications for the HapMap Project and disease association studies that rely on linkage disequilibrium. While haplotypes offer a shortcut for identifying candidate disease genes based on typing a certain number of markers, the number of markers required depends on the strength of linkage disequilibrium. If recombination rates differ across human populations, as these results suggest, then the strength of linkage disequilibrium will too—which means that association studies might need to adjust the number of markers needed to flag candidate disease genes in different populations.

